# Editorial: Editors' showcase: neuroplasticity and development

**DOI:** 10.3389/fnmol.2025.1558715

**Published:** 2025-03-06

**Authors:** Giovanni Cirillo, Michele Papa

**Affiliations:** Division of Human Anatomy - Neuronal Networks Morphology and Systems Biology Lab, Department of Mental, Physical Health and Preventive Medicine, University of Campania “Luigi Vanvitelli”, Naples, Italy

**Keywords:** neuroplasticity, BDNF, Cdk5, Arc/Arg3.1, AIS, neurodegenerative disorders

Synaptic plasticity, the ability of neurons to strengthen or weaken their connections in response to activity, is fundamental to learning, memory, and overall neural circuit function. This adaptability is orchestrated through intricate molecular and cellular mechanisms, including the regulation of local mRNA translation, post-translational modifications of key proteins, and cytoskeletal remodeling. The published studies of the proposed Research Topic provide valuable insights into different facets of neuronal plasticity, highlighting the molecular underpinnings that enable neurons to maintain flexibility while ensuring stability and functionality. Together, these studies illustrate the multifaceted mechanisms underlying neuronal adaptability, from localized mRNA translation and protein oligomerization to kinase signaling and cytoskeletal remodeling. By unraveling these processes, they enhance our understanding of both normal neuronal function and the pathophysiology of disorders like Alzheimer's disease, Huntington's disease, and other neurological conditions, paving the way for novel therapeutic strategies targeting neuronal plasticity.

Bimbi and Tongiorgi investigated the activity-dependent trafficking and local translation of brain-derived neurotrophic factor (BDNF) mRNA during chemical long-term potentiation (cLTP). BDNF, a neurotrophin critical for synaptic plasticity and neuronal development, plays dual roles as a locally synthesized protein and a modulator of synaptic maturation. Their research provided direct evidence of BDNF mRNA being locally transported and translated at activated synapses. By tracking the dynamics of BDNF mRNA and its protein product in mouse hippocampal neurons, they demonstrated that, within 15 min of cLTP induction, BDNF mRNA granules halt their movement near dendritic spines, forming a local reservoir for translation. By 30 min, these granules migrate into the spines, mirroring the behavior of other synaptic mRNAs such as CamkIIα. BDNF protein levels within the spines increase significantly after 60 min, indicating its role in late-phase synaptic strengthening. This study revealed a two-step mechanism of mRNA trafficking and translation, emphasizing the importance of spatial and temporal precision in linking protein synthesis to synaptic activation. These findings are critical for understanding how dysregulation in BDNF mRNA trafficking and translation may contribute to neurological conditions, including depression, schizophrenia, and Alzheimer's disease.

In a related domain, Hernández-Echeagaray et al. explored the role of cyclin-dependent kinase 5 (Cdk5) in modulating synaptic plasticity during the early stages of Huntington's disease (HD). Cdk5 is a serine/threonine kinase involved in various neuronal processes, including synaptic plasticity and survival. Using a 3-nitropropionic acid (3-NP) mouse model, which mimics mitochondrial dysfunction and neurodegeneration seen in HD, the researchers showed that subchronic 3-NP administration increased Cdk5 activity in the striatum without altering overall protein levels. This heightened activity correlated with deficits in corticostriatal synaptic plasticity, including impairments in long-term depression (LTD) and long-term potentiation (LTP). Notably, inhibition of Cdk5 with roscovitine restored LTP in medium spiny neurons (MSNs), provided dopamine D1 receptor signaling remained intact. This study highlighted the dual role of Cdk5, which acts both as a regulator of neuronal signaling and a contributor to synaptic dysfunction during early HD. Understanding these mechanisms opens pathways for therapeutic strategies that target Cdk5 to mitigate neurodegeneration and preserve synaptic flexibility.

Further exploring molecular regulators of synaptic plasticity, Mergiya et al. focused on Arc/Arg3.1, an immediate early gene product that plays a central role in synaptic plasticity. Arc facilitates long-term potentiation (LTP), long-term depression (LTD), and homeostatic scaling. The study investigated Arc's ability to form oligomeric complexes, a property that modulates its functional role in synaptic signaling. Using *in situ* protein crosslinking, the researchers identified Arc dimers as the predominant oligomeric form in the rat brain, with significant regional differences in their abundance. Synaptic activity, such as LTP induction in the dentate gyrus via high-frequency stimulation or BDNF infusion, increased Arc dimer levels. These dimers, captured by use of crosslinkers that stabilize non-covalent interactions, may contribute to rapid actions of Arc in regulating AMPA receptor trafficking and actin cytoskeletal dynamics, supporting changes in dendritic structure and synaptic function. The findings suggest that Arc dimers serve as molecular hubs for protein-protein interactions, facilitating the structural and functional adaptations necessary for learning and memory. The study positions Arc oligomerization as a potential target for therapeutic interventions aimed at mitigating synaptic dysfunction in neurological disorders.

Finally, Micinski and Hotulainen examined the structural plasticity of the axon initial segment (AIS), a critical neuronal domain responsible for maintaining polarity and generating action potentials. The study focused on actin cytoskeletal dynamics, particularly the role of actin polymerization and the formation of longitudinal actin fibers. Using super-resolution microscopy, they observed that depolarization-induced AIS plasticity caused a transient increase in longitudinal actin fibers within 3 h of stimulation, with these structures returning to baseline after 48 h. The study demonstrated that formin-mediated actin polymerization is essential for these changes, with the formin protein Daam1 specifically localizing to the ends of the longitudinal fibers. Inhibition of actin polymerization blocked both fiber formation and AIS remodeling, highlighting the critical role of cytoskeletal dynamics in AIS plasticity. The research proposed a stepwise mechanism involving actin ring destabilization, protein redistribution, and cytoskeletal re-stabilization. These findings underscore the interplay between structural and molecular mechanisms in regulating neuronal excitability and have implications for understanding conditions such as Alzheimer's disease, where AIS dysfunction is prevalent.

Together, these studies provide a comprehensive understanding of the diverse mechanisms underlying neuronal plasticity. BDNF mRNA trafficking exemplifies the precision of local translation in synaptic strengthening, with implications for the treatment of disorders characterized by synaptic dysregulation. Similarly, the modulation of synaptic plasticity by Cdk5 reveals the kinase's dual role as both a regulator and disruptor of neuronal signaling during neurodegeneration, underscoring its therapeutic potential in Huntington's disease. The investigation into Arc/Arg3.1 oligomerization expands knowledge of how protein complexes orchestrate synaptic remodeling, offering insights into learning and memory processes. Lastly, the research on AIS plasticity highlights the critical role of cytoskeletal dynamics in maintaining neuronal excitability, with broader implications for understanding and treating neurological conditions involving structural dysfunction. These findings collectively highlight the intricate interplay of molecular, structural, and signaling mechanisms that enable neurons to adapt to dynamic physiological and pathological conditions. They also emphasize the importance of continued research into these processes to develop targeted therapies for a range of neurological disorders. By elucidating the fundamental principles of neuronal plasticity, these studies pave the way for innovative strategies to enhance cognitive function and mitigate the effects of neurodegeneration.

